# Unveiling the conserved mechanism of microsporidian vertical transmission: A comparative study of *Nosema* infection across host species

**DOI:** 10.1080/21505594.2025.2609384

**Published:** 2025-12-23

**Authors:** Chunxia Wang, Yongzhi Kong, Zishen Tang, Tongyu Luo, Xuanang Yang, Yongjun Zhang, Tian Li, Zeyang Zhou

**Affiliations:** a State Key Laboratory of Resource Insects, Southwest University, Chongqing, China; b Chongqing Key Laboratory of Microsporidia Infection and Control, Southwest University, Chongqing, China; c Sericultural Research Institute of Henan Province, Zhengzhou, China; d College of Life Sciences, Chongqing Normal University, Chongqing, China

**Keywords:** Transovarial transmission, microsporidia, silkworm, mechanism, vitellogenin

## Abstract

Microsporidia, ubiquitous obligate intracellular parasites infecting a wide range of hosts from humans to economically vital animals, employ transovarial transmission (TOT) as their primary vertical transmission mode. Despite its significance, the mechanisms underpinning microsporidian TOT have remained elusive. This study comparatively analyzed the TOT in two distinct systems: *Nosema pernyi* infecting wild tussah *Antheraea pernyi*, and *Nosema bombycis* infecting domestic silkworms *Bombyx mori* and crop pests *Spodoptera litura* and *Helicoverpa armigera*. Our findings reveal that both parasites share a conserved invasion sequence targeting ovariole sheath cells, follicular cells, nurse cells, and ultimately oocytes. Notably, infection of follicular and nurse cells consistently precedes oocyte invasion, suggesting a strategic utilization of these cells for efficient transmission. Contrasting patterns were observed between the two parasites: while *N. bombycis* exhibits lower infection rates and produces mature spores in both oocytes and nurse cells, *N. pernyi* displays higher parasite loads with proliferative stages predominant throughout infection. A critical innovation emerges from our RNA interference experiments, where knockdown of host vitellogenin (Vg) significantly reduced microsporidian loads, identifying Vg as a conserved molecular facilitator in TOT. These findings not only elucidate the evolutionary conservation of vertical transmission mechanisms among microsporidia but also pinpoint Vg as a promising target for intervention against these pathogens. This research advances our understanding of vertical transmission of fungal parasites and offers novel avenues for disease control.

## Introduction

Microsporidia are a group of obligate intracellular eukaryotic parasites that inflict substantial economic losses on agriculture and threaten human health by causing various diseases [1–5]. Microsporidia are widely distributed in nature, and more than 220 genera and 1700 species have been identified [6–8]. *Nosema bombycis* is the first identified microsporidium and causes domestic silkworm pébrine disease [9,10]. In addition, *Nosema pernyi* is a pathogen infecting the Chinese oak silkworm, *Antheraea pernyi*, also leading to pébrine disease [11–14]. In general, microsporidia mainly infect hosts through horizontal transmission. Some can also be transmitted horizontally and vertically, while the minority can only be transmitted vertically. Vertical transmission is common in the viruses, bacteria, protists, and helminths [15,16], mainly including transovarial transmission (TOT) and transplacental transmission [16,17]. TOT is a minor route supplementary to the main horizontal route in microsporidia. However, for a large subset of arthropod-infecting microsporidia, TOT is crucial for parasite maintenance in the host population, with transmission strategies including alternating between horizontal and transovarial routes [18,19]. The parasites, *N. bombycis* and *N. pernyi*, can be transmitted transovarially to the next generation through oocyte infection. Due to its capability for TOT, microsporidian *N. bombycis* and *N. pernyi* pose a substantial threat to the sericulture industry [20–23]. Our recent research has revealed the main process and a mediating factor, vitellogenin (Vg), of *N. bombycis* TOT in the silkworm *Bombyx mori* [24]. However, it remains unclear how microsporidia in different hosts infect the oocyte.

It has been established that the TOT mechanism is a prevalent and critical strategy employed by numerous pathogens, including viruses, bacteria, and microsporidia [15,16,25]. The lepidopteran insect ovary consists of multiple ovarioles, each of which comprises a germarium, vitellarium, and pedicel extending from the apex to the base. Oocytes generated in the germarium are arranged linearly within the vitellarium and enveloped by a layer of follicular cells [17,26]. The establishment of TOT is primarily dependent on the capacity of pathogens to infect host germ cells, as different pathogens exhibit varying degrees of infectivity toward host ovariole cells. Research has demonstrated that different pathogens, such as rice stripe virus (RSV) and *Wolbachia*, exhibit a consistent infection pattern within germ cells of the same host species, exemplified by *Laodelphax striatellus*, where they infect follicular cells, trophocytes, and oocytes [27,28]. *Rickettsia* can infect the early developing oocytes and follicular cells to achieve vertical transmission [29]. Numerous studies have documented microsporidian infections in the reproductive tissues and eggs of hosts [30–35]. For example, the microsporidian *Nosema heliothidis* and *Nosema plodia* infect host nurse cells and proliferate within them before being transferred to oocytes [30,31]. Moreover, our recent study elucidates the process by which *N. bombycis* invades oocytes from the hemolymph, encompassing the infection of ovariole sheath cells, follicular cells, and nurse cells [24]. It has been demonstrated that *N. bombycis* also infects follicular cells, nurse cells, and oocytes for TOT in crop pests *Spodoptera litura* and *Helicoverpa armigera* [36]. *N. pernyi* infects the midgut epithelium, silk glands, muscle tissues, fat body, blood cells, and reproductive organs of *A. pernyi* [37–41]. However, there have been no reported studies on the infection of host reproductive cells of *N. pernyi*. It is worth investigating whether *N. pernyi* exhibits infectivity toward host germ cells that is similar to that of other *Nosema* species, and whether *Nosema* parasites generally utilize similar strategies to infect host germ cells.

For successful transmission to offspring, pathogens must traverse multiple protective barriers to access the oocytes. The infection of host oocytes by the pathogen is a prerequisite for vertical transmission, and the timing and mode of oocyte invasion by microorganisms vary, with diverse strategies documented [16,19,28,30,42–44]. For instance, RSV enters the nurse cells of the germarium via endocytosis and subsequently migrates to the oocytes of *L. striatellus* through nutritive cords [28]. Similarly, rice gall dwarf virus (RGDV) utilizes virus-containing tubules to traverse follicular cells, thereby overcoming TOT barriers [44]. Within follicular cells and nurse cells of *B. mori*, *N. bombycis* undergoes restructuring and forms large vacuoles for delivering parasites into the oocytes. However, the specific mechanism of this process still requires further investigation, and whether it constitutes a conservative transmission route also needs verification.

The molecular mechanisms underlying TOT have been investigated in several pathogens. Current evidence indicates that viruses exploit preexisting pathways utilized by vitellogenin (Vg), a major yolk protein precursor essential for oocyte development in insects, to gain entry into oocytes [17,45]. Vg functions extend beyond serving as a nutrient reserve for embryonic development. Its domains contribute not only to yolk formation in oocytes but also to pathogen recognition [46–49]. The evolutionary conservation and functional importance of Vg may enable microorganisms to exploit it for vertical transmission. For example, RSV and tomato yellow leaf curl virus (TYLCV) can directly interact with vector-derived Vg, which serves as a vehicle facilitating their entry into the oocyte [25,28,50]. Similarly, endosymbiotic bacteria such as *Spiroplasma poulsonii* in *Drosophila melanogaster* and *Wolbachia* in *L. striatellus* can utilize host Vg/Vg receptor-mediated endocytosis to achieve TOT [27,42]. This symbiotic bacterium targets oocytes by utilizing existing host molecules and cellular mechanisms, this is the result of long-term coevolution between the insect host and the symbiotic microorganism. To reach the host ovariole and oocyte, pathogens widely exploit this conserved transport system for TOT. Moreover, *N. bombycis* interacts directly with Vg during TOT not only in *B. mori* but also in *S. litura* and *H. armigera* [24]. Little research has been conducted on the specific mechanism of the TOT process in microsporidia. Therefore, it is of critical importance to investigate the common mechanisms underlying TOT among different parasites across hosts.

In this study, we conducted comprehensive investigations into how *Nosema* species infect host oocytes through TOT. By comparing the infection processes and underlying mechanisms of *N. pernyi* infecting *A. pernyi* and *N. bombycis* infecting *B. mori*, *S. litura*, and *H. armigera*, respectively, we aimed to elucidate both the conserved and divergent strategies employed by microsporidia in their TOT. This comparative approach provides deeper insights into the evolutionary and adaptive strategies of microsporidian vertical transmission, thereby shedding light on the factors influencing parasite virulence and their survival across different host species.

## Materials and methods

### Insects rearing and infection

Chinese oak silkworm *A. pernyi* samples (fifth late-instar larvae and pupae) were collected from the Sericultural Research Institute of Henan Province in China. The pupae were kept in a rearing chamber at 28°C and injected with 1 × 10^8^ *N. pernyi* spores per pupa for infection. The infected pupae were examined by microscopic observation and used to extract *N. pernyi* parasite spores and collect ovaries and ovarioles at different developmental stages to observe the infection.

The domestic silkworms, *B. mori* Guican No. 5 were reared with an artificial diet at 26°C, 70% relative humidity, and 12 h light and dark [51]. The fourth-instar domestic silkworms were individually orally inoculated with *N. bombycis* spores at a concentration of 1 × 10^4^ spores per larva, and normal feeding was subsequently performed until pupation. Infected ovaries and ovarioles on the first to fifth day after pupation were obtained for subsequent experiments.

The *S. litura* and *H. armigera* were purchased from Jiyuan Baiyun Industrial Co., Ltd., Henan, China. The larvae were reared with a fresh artificial diet at 26°C, 70% relative humidity, and 12 h light and dark [36]. The third-instar larvae of *S. litura* and *H. armigera* were fed with 2 × 10^3^ *N. bombycis* spores per larva for infection. Normal feeding was subsequently performed until pupation, from which the infected ovariole tissues were isolated for subsequent experiments.

### Microscopic observation of the pupae tissues

The midgut, fat body, malpighian tubules, ovary, testis, and hemolymph of the pupae were collected and observed using an OLYMPUS SZX16 (Olympus, Tokyo, Japan) with a 1× objective lens and a 10× eyepiece, and photographed using an OLYMPUS cellSens Standard 1.18. The infected tissue slides were directly observed using OLYMPUS B×53 with a 100× objective lens, and a 10× eyepiece and photographed with cellSens Dimension 1.6.

### Microsporidia spore purification, sequencing, and phylogenetic analysis

Infected Chinese oak silkworm pupae were collected for spore purification. The infected pupal tissue was homogenized in sterilized water and filtered using thick cotton in a 10 mL centrifuge tube. The remaining liquid was centrifuged at 3000 rpm for 5 min, and the pellet was washed three times and resuspended in distilled water. The spores were purified by centrifugation at 10,000 × g for 15 min in 75% Percoll (17089101, Cytiva, USA). The purified spores were washed three times with sterilized water and stored at 4°C for use. The purified spores were examined using a light microscope (Olympus, Tokyo) and photographed with cellSens Dimension 1.6.

Then, the DNA of purified spores (5 × 10^8^) was extracted using the CTAB method. Specifically, 200 μL of spore suspension was processed for genomic DNA extraction by adding 500 μL of CTAB buffer and 25 μL of protease K, followed by incubation at 56°C for 2 h. The subsequent extraction procedures followed the previously described protocol [52,53]. The extracted genomic DNA of microsporidia was used as a template to perform PCR amplification with the small subunit (SSU) rRNA gene (Table S1). The PCR reactions were performed in 50 µL final reaction volumes containing 1 µL of each primer, 25 µL Prime STAR Max DNA Polymerase, 22 µL nuclease-free water, and 1 µL diluted gDNA template. Amplification was carried out in a thermal cycler using an initial denaturation at 98°C for 2 min, followed by 30 cycles of denaturation at 98°C for 10 s, annealing at 55°C for 15 s, and extension at 72°C for 15 s. The PCR products were eluted by an E.Z.N.A. Gel Extraction Kit (Omega), and the products were connected with pMD19-T vector and transferred into Trans5α, and the positive clones were screened and sent to Sangon Biotech (Shanghai, China) for sequencing. The sequencing results were submitted to NCBI for BLAST homology comparison, and the phylogenetic analysis of microsporidia was performed using the neighbor-joining method with MEGA 11.0 [54].

### Antibody detection by Immunofluorescence assay and western blotting

Immunofluorescence assay and western blot assay were used to detect whether anti-*N. bombycis* can recognize *N. pernyi*. *N. pernyi* spores and *N. bombycis* spores were washed and fixed. They were subsequently blocked with tyrosine buffer and incubated with anti-*N. bombycis* (1:200 dilution) for 90 min at 37°C, washed three times with PBST (0.01 M PBS +0.05% Tween 20) each for 5 min, followed by incubation with goat anti-rabbit secondary antibody labeled with Alexa Flour 488 (A32731, Invitrogen, USA), fluorescent brightener 28 (FB 28) (ZY4404, China) for staining chitin, and propidium iodide (PI) (P3566, Invitrogen) for staining nuclei in a dark environment for 45 min. The slides were washed three times with PBST, then suspended in Fluoromount Aqueous Mounting Medium (F4680, Sigma) cover glass.

The fat body infected by *N. pernyi* and fat body infected by *N. bombycis* were ground in liquid nitrogen, and the proteins were extracted using lysis buffer (20 mM Tris, pH 7.4, 0.15 M NaCl, 1 mM EDTA, 0.1% Triton-X, 0.1% sodium dodecyl sulfate). *N. pernyi* spores and *N. bombycis* spores were disrupted 5 min for three times with 0.4 g acid-washed glass beads (0.2 g, 212–300 μm; 0.2 g, 425–600 μm; Sigma-Aldrich) in 500 μl buffer PBS (pH 7.4). The samples were centrifuged at 13,000× *g* for 30 min, the supernatant collected, and the protein concentration quantified using the bicinchoninic acid protein assay (P0010S, Beyotime). For immunoblotting analysis, the total protein samples and the ladder (26616, Thermo Fisher Scientific) were separated using 10% sodium dodecyl-sulfate polyacrylamide gel electrophoresis (SDS-PAGE) and transferred to a polyvinylidene difluoride (PVDF) membrane (Roche, Switzerland). The membrane was incubated with anti- *N. bombycis* (1:500) for 90 min at 37°C. Thereafter, it was washed three times with Tris-buffered saline containing 0.05% Tween 20 (TBST), and incubated for 1 h at 37°C with HRP-linked anti-rabbit IgG antibody (BL001A, Bioshap, China). After three washes with TBST, the membrane was exposed to an ECL western blot detection kit (34580, Thermo Fisher Scientific) and imaged using the ChemiScope 6200 Touch (Clinx, China). The experimental operation for detecting whether antibody of vitellogenin (Vg) of *B. mori* can specifically recognize the Vg of *A. pernyi* is the same as the above method.

### Paraffin section and indirect immunofluorescent assay (IFA)

The normal and infected ovarioles were prepared for paraffin section. The paraffin embedding and sectioning of the samples were carried out as described previously [23,24]. After deparaffinization and hydration, the slices were incubated with antigen repair solution and stored at 98–100°C for 20 min, and then incubated with polyclonal antibody against *N. bombycis* at room temperature for 90 min and washed three times with PBST (0.01 M PBS +0.05% Tween 20) each for 5 min, followed by incubation with goat anti-rabbit secondary antibody labeled with Alexa Flour 488, FB 28 for staining chitin, and PI for staining nuclei in the dark for 50 min. The slides were suspended by Fluoromount Aqueous Mounting Medium and mounted with a cover glass after washing three times with PBST. The samples were observed and photographed using the OLYMPUS Biological Confocal Laser Scanning Microscope FV1200 (Olympus, Tokyo) with 200× and 1000× magnifications for global and local observations, respectively.

### Transmission electron microscopy

The purified *N. pernyi* spores and the ovarioles (as described above) infected with *N. pernyi* were fixed in 2.5% glutaraldehyde immediately after excision. The fixed samples were washed in 0.1 M PBS (pH 7.4) four times (15 min each) and postfixed in 1% osmium tetroxide for 2 h, followed by four additional washes (10 min each) in 0.1 M PBS. Thereafter, the samples were dehydrated using an ascending ethanol series and 100% acetone, infiltrated with gradient Epon812 (SPI, USA) resin, sequentially embedded in absolute resin, and polymerized at 70°C for 48 h. A Leica EM UC7 ultramicrotome (Leica, Germany) was used to acquire ultrathin sections, which were placed on nickel grids and stained with 3% uranyl acetate, followed by lead citrate. The stained grids were rinsed six times with ddH_2_O, dried, and photographed using a JEM-1400 Plus transmission electron microscope (JEOL, Japan) at an acceleration voltage of 80 kV.

### Detecting the localization of Vg with parasites

In order to detect the binding between Vg and spores in infected *A. pernyi*, spores were isolated from the pupae, washed, and fixed. They were subsequently blocked with tyrosine buffer and incubated with anti-Vg (1:100 dilution) for 2 h at 37°C, followed by incubation with Alexa 488-labeled secondary antibody, and 4,6-diamidino-2-phenylindole (DAPI) (D9542, Sigma) for detecting nuclei. The same sample was also used for the western blotting. The operation was as previously described above.

To observe the colocalization of Vg and parasite in the ovarioles of *A. pernyi*, *B. mori*, *S. litura*, and *H. armigera*, the sections were incubated with rabbit anti-*N. bombycis* polyclonal antibody (1:200), mouse anti-Vg polyclonal antibody (1:200) at 37°C for 90 min and washed three times with PBST each for 5 min, followed by incubation with goat anti-rabbit secondary antibody labeled with Alexa Flour 488, goat anti-mouse secondary antibody labeled with Alexa Flour 647 (A32728, Invitrogen) and washed three times with PBST for 5 min each. All the antibodies used in the experiment were obtained from previous studies [24]. Finally, the sections were incubated with a mixed solution of FB 28 and PI to stain the chitin and nuclei, respectively.

Detecting the binding of spores with Vg domain VWD and the colocalization of Vg domain VWD with SWP12, SWP26, and SWP30. *S. litura* vitellogenin VWD domain and *H. armigera* vitellogenin VWD domain gene sequences were synthesized by Sangon Biotech (Shanghai, China) and cloned into the vector pET-30a (+) for protein purification, and the experimental procedures for vector construction and protein purification were all carried out in accordance with the previous methods [24]. To confirm the binding of spores with Vg domain VWD, we firstly incubated spores with purified recombinant protein (rSlVgVWD and rHaVgVWD), and then detected by IFA. At the same time, after incubating spores with purified recombinant protein, we then detected the colocalization of recombinant proteins (rSlVgVWD and rHaVgVWD) with SWP12, SWP26, and SWP30 on the spore surface using IFA. Vg was labeled with secondary antibodies coupled with Alexa Fluor 488. SWP12, SWP26, and SWP30 were stained using a secondary antibody coupled with Alexa Fluor 594 (A32740, Invitrogen), and the spore nuclei were stained with DAPI. Finally, the samples were observed and photographed using an Olympus Biological Confocal Laser Scanning Microscope FV1200.

## RNAi of ApVg and qPCR analysis

We then used RNAi strategy to determine the functional roles of vitellogenin (AB049631) during the *N. pernyi* infection in the pupal stage. Briefly, dsRNAs targeting 472 bp regions of ApVg, and green fluorescent protein (GFP) genes were synthesized using the T7 RiboMAX Express RNAi System (P1700, Promega, USA) according to the manufacturer’s instructions. The specific primers used to generate the DNA templates are listed in Table S1. The pupae of oak silkworm in diapause stage were injected with 1 × 10^8^ *N. pernyi* spores to obtain pupae with equal parasites abundance in hemolymph. The acquisition, injection dose, and injection process of double-stranded RNA (dsRNA) were performed as previously described with minor modifications [24]. Female oak silkworms on the pupal stage were injected with 20 μg of dsApVg three times. An equal volume of dsGFP was simultaneously injected as a control. Thereafter, the fat bodies of pupae were collected for the detection of ApVg transcripts and expression, respectively.

Total RNA was extracted using the EZNA™ Total RNA Kit II (R6934-01, OMEGA, USA) according to the manufacturer’s instructions. The cDNA was synthesized with 1 μg total RNA using the GoScript™ Reverse Transcription System Kit (A5003, Promega) after DNA digestion with DNase I. Relative ApVg mRNA levels were measured using qPCR with specific primers and 18S ribosomal RNA gene (DQ347469) primers (Table S1) as follows: a pre-denaturation of 95°C for 2 min, followed by 40 cycles at 95°C for 10 s, and 60°C for 20 s. The relative gene expression level was calculated using the 2^−∆∆Ct^ method. The protein expression levels of ApVg in fat bodies and ovarioles were detected by western blotting after total protein extraction, and tubulin was used as a reference according to the method of appeal experiment.

To investigate the *N. pernyi* infection status of ovarioles after RNAi, Genomic DNA was extracted from the samples using the EZNA™ Tissue DNA Kit (D3396-02, OMEGA) according to the manufacturer’s instructions. The Genomic DNA was used as the template, and the initial copy number of *N. pernyi SSU* was calculated using qPCR with specific primers (Table S1). A known number of copies (1.0 × 10^8^) of the recombinant pMD™ 19-T Vector (6013, TaKaRa) plasmid with *N. pernyi SSU* was used as the standard. qPCR was performed follows: a pre-denaturation of 95°C for 2 min, followed by 40 cycles at 95°C for 10 s and 60°C for 20 s. The infection rate was calculated as the *N. pernyi SSU* copy number ratio between the experimental and control groups.

## Results

### The infection of *A.* *pernyi* pupae by *N.* *pernyi*

**Figure 1.**
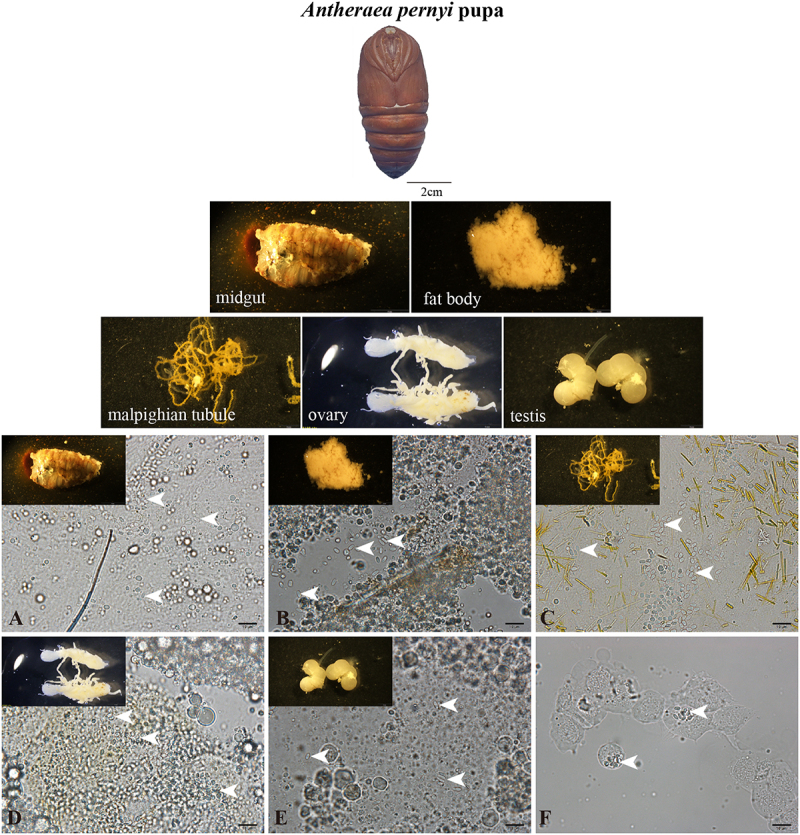
Microscopy observation of microsporidian infections in *A. pernyi* pupae tissues. (A) Midgut, (B) fat body, (C) Malpighian tubules, (D) ovary, (E) testis, and (F) hemolymph. The bar indicates 10 µm. The arrowhead shows the microsporidia spore.

**Figure 2.**
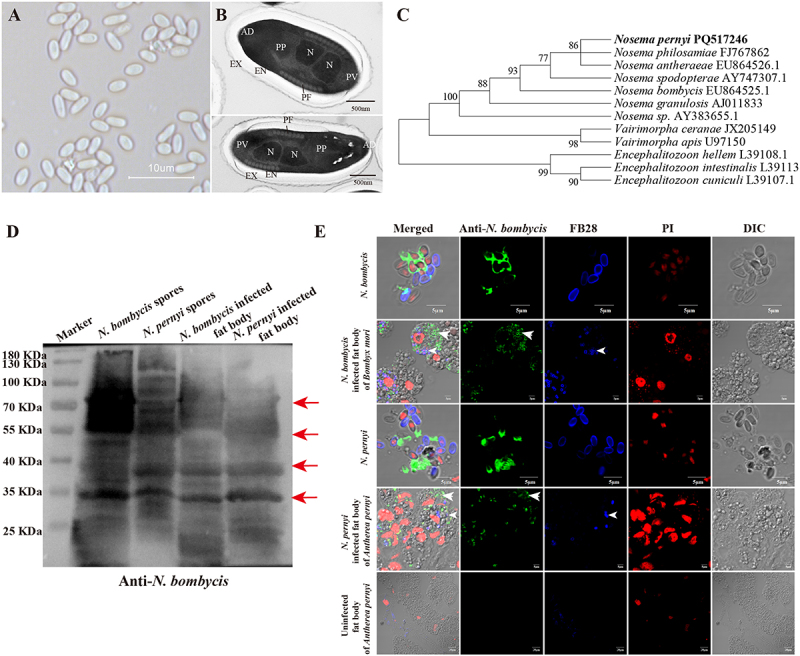
Characteristics of *N. pernyi* spore and antibody verification. (A) Microscopy observation of microsporidian spores isolated from *A. pernyi*. (B) TEM micrograph of *N. pernyi* spores, showing the exospore (EX), endospore (EN), nucleus (N), polar filament (PF), anchoring disc (AD), anterior region (AP), polaroplast (PP), and posterior vacuole (PV). (C) Phylogenetic tree of microsporidia isolated from *A. pernyi* and related species constructed by neighbor-joining method based on *SSU* rRNA sequences. (D) Detection of *N. pernyi* spores using polyclonal antibodies against anti-*N. bombycis* using western blotting, red arrow indicates the primary band of the antibody recognition. (E) Detection of *N. pernyi* spores in tissues using polyclonal antibodies against anti-*N. bombycis* using IFA. Spores were stained using FB28 (blue); proliferative *N. pernyi* were labeled with rabbit polyclonal antibody against *N. bombycis* (Alexa488, green); nucleus stained by PI (red).

All examined *A. pernyi* pupal tissues were found to be infected, including the midgut (Figure 1A), fat body (Figure 1B), malpighian tubules (Figure 1C), ovary (Figure 1D), testis (Figure 1E), and hemolymph (Figure 1F). *N. pernyi* spores were 3.68 ± 0.47 and 2.09 ± 0.52 μm in length and width, respectively (Figure 2A) and were diplokaryotic nuclei, which were encircled by 9–12 coils of the polar tube (Figure 2B). The phylogenetic tree constructed using SSU rRNA sequences indicated that *N. pernyi* belongs to the *Nosema* genus and has a closer relationship with *Nosema antheraeae* and *Nosema philosamiae* (Figure 2C). Moreover, as shown in Figure 2D, E, *N. pernyi* purified and in the infected tissues could be labeled by a polyclonal antibody primarily produced by inoculating mice with *N. bombycis* [23,24].

### *N.* *pernyi* infects the ovarian wall and ovarioles at the diapause pupal stage

**Figure 3.**
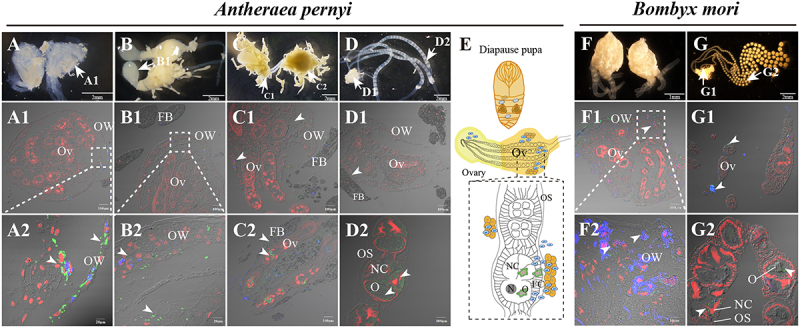
Ovaries and ovarioles were infected by parasites. Paraffin sections of the infected ovaries and ovarioles in the prepupal stage (A), Diapause pupal stage (B, C), and non-diapause pupal stage (D) were prepared. (A1–D1 and A2–D2) Immunofluorescence assay on the paraffin sections of the infected *A. pernyi* ovaries and ovarioles. (E) The model of the *A. pernyi* ovary infected by *N. pernyi* during diapause stage. *N. pernyi* spores stained with FB28 (blue); *N. pernyi* proliferative parasites labeled with antibody conjugated to Alexa Fluor 488 (green). Immunofluorescence assay on the paraffin sections of the infected *B. mori* ovaries on the first day of pupation (F) and ovarioles on the fourth day of pupation (G). *N. bombycis* spores stained with FB28 (blue); *N. bombycis* proliferative parasites labeled with antibody conjugated to Alexa Fluor 488 (green); nucleus stained by PI (red). OW, ovarian wall; OV, ovariole; OS, ovariole sheath; FB, fat body; NC, nurse cell; O, oocyte; arrowhead shows the parasites infection.

In the diapause stage, the ovaries are rod-shaped, with the head constituting the primary part of the ovary, and the exposed ovariole at the tail being tightly enclosed by the fat body (Figure 3A,C). Once the pupa has been relieved from diapause, the ovariole extends into the hemolymph to initiate follicle development (Figure 3D). As the follicles develop, the ovariole absorbs nutrients, causing the egg to enlarge and ultimately form a mature egg that is subsequently laid by the moth.

Immunostaining analysis of the paraffin sections of ovaries at pre-pupal stage revealed the infection of ovarian wall cells (Figure 3A,B). Further examination of ovaries at the diapause stage showed infections not only in ovarian wall cells but also within the interior of the enclosed ovarioles (Figure 3C), which were fully infected after diapause was released (Figure 3D). Notably, the tail of the rod-shaped ovary and the surrounding fat bodies were heavily infected by *N. pernyi* (Figure 3C2). In contrast, *N. bombycis* is unable to invade the ovariole in *B. mori*, until ovariole breaches through the ovarian wall and becomes exposed to hemolymph at the pupal stage (Figure 3F1–G2). These observations suggest that the timing of ovariole invasion differs significantly from that in *N. bombycis* infection, as *N. pernyi* is capable of invading the ovarioles during the extended diapause pupal stage (Figure 3E).

### *Nosema* parasites infect the ovariole sheath, follicular cells, and nurse cells

**Figure 4.**
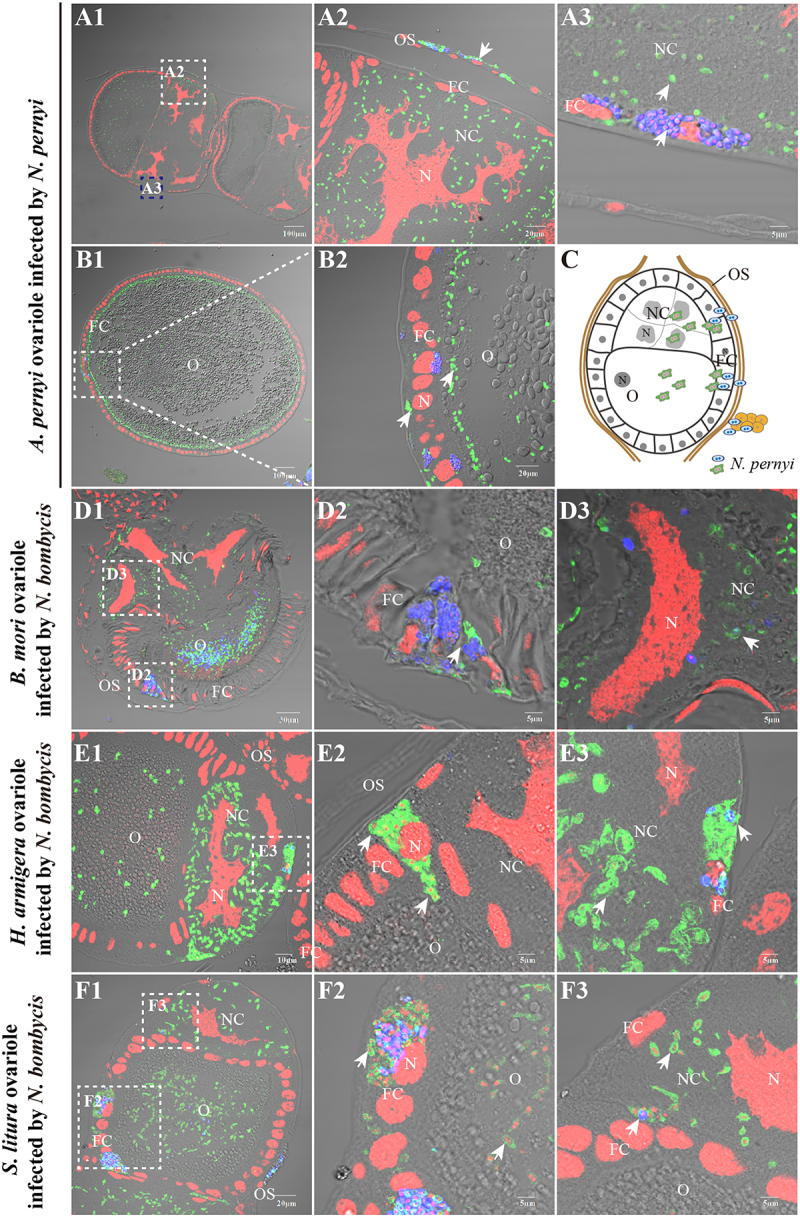
Paraffin sections of ovarioles infected by parasites. (A1) The infected *A. pernyi* ovarioles in the non-diapause stage. (A2–A3) enlarged views of the infected ovariole sheath cells and follicular cells. (B–B1) The infected follicular cells and oocyte. (C) The model of the *A. pernyi* ovarioles infected by *N. pernyi*. (D1–D3) *N. bombycis* distribution in the infected *B. mori* follicles. (E1–E3) *N. bombycis* distribution in the infected *H. armigera* follicles. (F1–F3) *N. bombycis* distribution in the infected *S. litura* follicles. The spores of parasites were stained with FB28 (blue); the proliferative parasites were labeled with antibody conjugated to Alexa Fluor 488 (green); nucleus stained by PI (red). OS, ovariole sheath; FB, fat body; NC, nurse cell; O, oocyte. Arrows show the parasites infection.

Observations on the ovarioles that were released from diapause showed that the infection began to penetrate into the internal cells, from ovariole sheath cells to follicular cells, nurse cells (Figure 4A) and oocytes (Figure 4B1–B2). Similar infection patterns (Figure 4C) were also observed in the ovarioles of *B. mori* (Figure 4D1–D4), *H. armigera* (Figure 4E1–E4), and *S. litura* (Figure 4F1–F4) following infection with *N. bombycis*. This conserved pattern of infection suggests that microsporidia may utilize similar cellular targets and mechanisms to establish vertical transmission across diverse insect species.

### *Nosema* parasites invade oocytes from follicular cells and nurse cells

**Figure 5.**
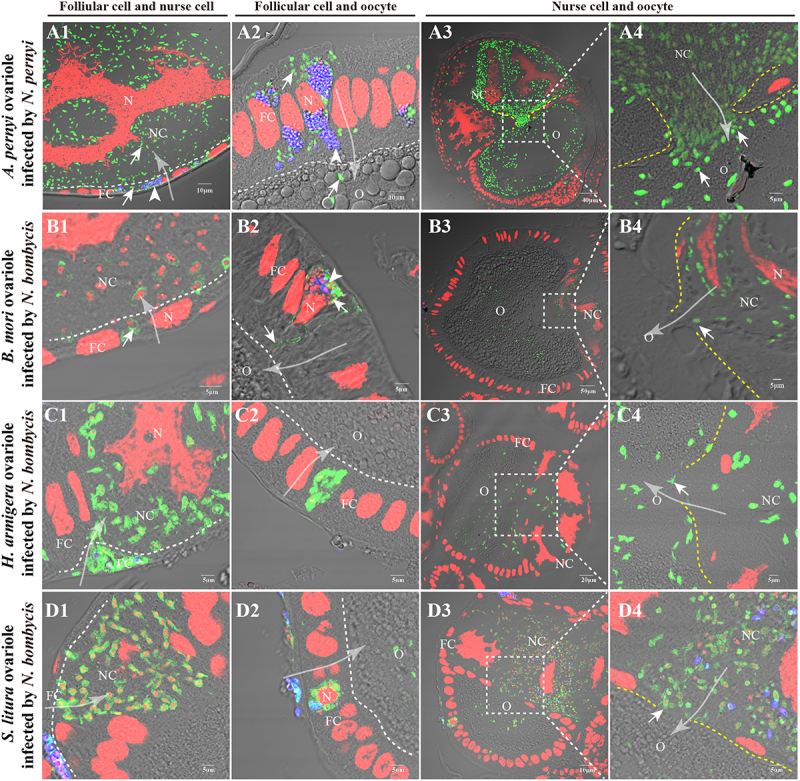
Parasites invaded oocytes from the follicular cells and nurse cells. (A1–A4) IFA showed that *N. pernyi*-infected oocytes in the *A. pernyi* follicles. (A1) *N. pernyi* invaded nurse cells from follicular cells. (A2) *N. pernyi* invaded oocytes from follicular cells. (A3–A4) *N. pernyi* invaded oocytes from the nurse cells. (B1–B4) IFA showed that *N. bombycis* infected oocytes in the *B. mori* follicles. (B1) *N. bombycis* invaded nurse cells from follicular cells. (B2) *N. bombycis* invaded oocytes from follicular cells. (B3–B4) *N. bombycis* invaded oocytes from the nurse cells. (C1–C4) IFA showed that *N. bombycis* infected oocytes in the *H. armigera* follicles. (C1) *N. bombycis* invaded nurse cells from follicular cells. (C2) *N. bombycis* invaded oocytes from follicular cells. (C3–C4) *N. bombycis* invaded oocytes from the nurse cells. (D1–D4) IFA showed that *N. bombycis* infected oocytes in the *S. litura* follicles. (D1) *N. bombycis* invaded nurse cells from follicular cells. (D2) *N. bombycis* invaded oocytes from follicular cells. (D3–D4) *N. bombycis* invaded oocytes from the nurse cells. The white dashed lines indicate the boundaries of cells; the big white arrows indicate the direction in which the parasites enter the oocyte from follicular cells or nurse cells. Spores of parasites were stained with FB28 (blue); the proliferative parasites were labeled with antibody conjugated to Alexa Fluor 488 (green); nucleus stained by PI (red); FC, follicular cell; O, oocyte; NC, nurse cell. Arrowhead shows mature spores; arrow shows proliferative parasites.

After invading the ovariole sheath cells, *N. pernyi* can subsequently spread to adjacent follicular cells (Figure 5A1,A2). Following proliferation within the follicular cytoplasm, they then invade nurse cells (Figure 5A1) and oocytes (Fig A2). Alternatively, the parasites located in nurse cells probably access oocytes through the nutrient delivery pathway utilized by nurse cells (Fig A3-A4). Notably, we only observed parasites in proliferative stage in the tunnel to oocyte, instead of mature spores. Our observations revealed that the parasite infect oocyte of *B. mori* (Figure 5B1–B4), *H. armigera* (Figure 5C1–C4), and *S. litura* (Figure 5D1–D4) by same routes, underscoring the conservation of microsporidian TOT. In general, the parasites enter oocytes via two primary pathways: (1) Follicular Cell Invasion Pathway: parasites initially invade follicular cells, proliferate within them, and subsequently invade nurse cells and oocytes; (2) Nurse Cell Invasion Pathway: parasites located in nurse cells utilize the nutrient delivery pathway to access and infect oocytes.

**Figure 6.**
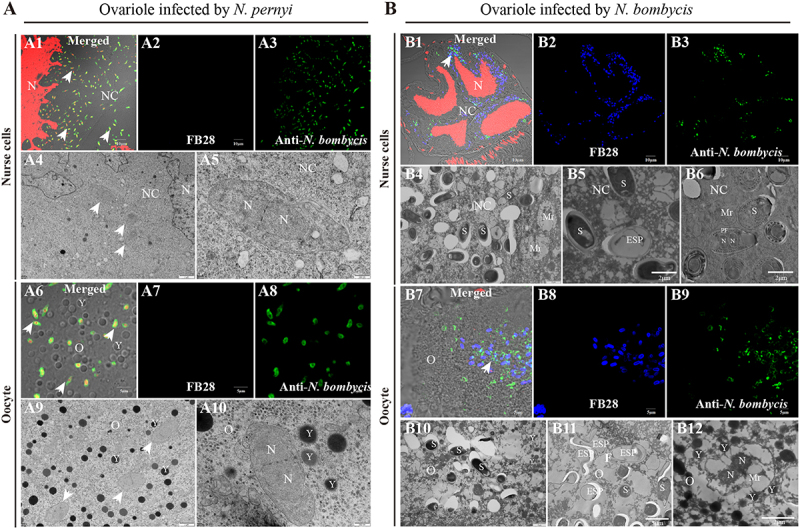
Characteristics of parasites within the ovariole cells. (A) characteristics of *N. pernyi* parasites within the *A. pernyi* ovariole cells. (A1–A3) IFA showed the *N. pernyi* infection in nurse cells. (A4–A5) TEM observation of infected nurse cell. (A6–A8) IFA showed the *N. pernyi* infection in oocyte. (A9–A10) TEM observation of infected oocyte. (B) Characteristics of *N. bombycis* parasites within the *B. mori* ovariole cells. (B1–B3) IFA showed the *N. bombycis* infection in nurse cells. (B4–B6) TEM observation of infected nurse cell. (B7–B9) IFA showed the *N. bombycis* infection in oocyte. (B10–B12) TEM observation of infected oocyte. Spores were stained using FB28 (blue); proliferative parasites were labelled with rabbit polyclonal antibody against *N. bombycis* (Alexa488, green); nucleus stained by PI (red); N, Nucleus; NC, nurse cell; O, oocyte; Y, yolk granules; S, spore; ESP, empty spore shell; Mr, meront; Arrow shows the parasite proliferation.

The parasites continuously replicate and proliferate in ovariole, eventually occupying the entire cell. We observed mature spores of *N. pernyi* exclusively in the cells of the ovariole sheath (Figure 4A2) and follicular cells (Figure 5A2). No spore-shaped parasites were detected at the junction between follicular cells and nurse cells, or within the nurse cells (Figure 6A1–A4) and oocytes (Figure 6A6–A9). In contrast, *N. bombycis* in forms of meronts, mature spores, and empty-shell spores were observed in the nurse cells and oocytes (Figure 6B1–B12). In addition, we observed that the two parasites displayed distinct distribution patterns following their entry into the oocyte. *N. bombycis* was uniformly distributed throughout the cytoplasm of oocyte, whereas *N. pernyi* was predominantly enriched in the inner region adjacent to the oocyte membrane (Fig S1). These findings suggest that both *N. bombycis* and *N. pernyi* likely penetrate the oocyte membrane barrier mainly in their proliferative stages instead of in mature spores.

### *Nosema* parasites preferentially infect nurse cells within the ovarioles to gain access to oocytes

**Figure 7.**
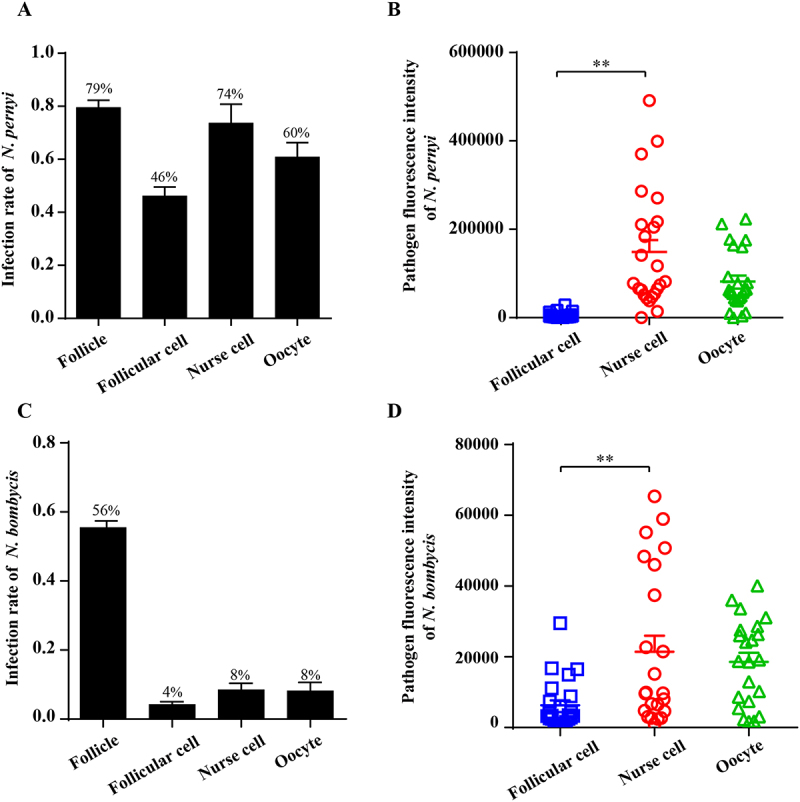
Distribution of parasites within the ovariole cells. (A) The *N. pernyi* infection rate in the *A. pernyi* follicles. (B) The fluorescence intensity shows the pathogen fluorescence intensity of *N. pernyi* in follicular cells, nurse cells and oocyte. (C) The *N. bombycis* infection rate in the *B. mori* follicles. (D) The fluorescence intensity shows the pathogen fluorescence intensity of *N. bombycis* in follicular cells, nurse cells and oocyte. ** *p* < 0.01, *n* = 25.

We further analyzed the infection rate and distribution patterns of the parasites in ovariole cells, in which infected by *N. pernyi* the follicle infection rate was 79%, follicular cell infection rate was 46%, nurse cell infection rate was 74%, and oocyte infection rate was 60% (Figure 7A). In comparison, the infection rates of *B. mori* ovariole cells by *N. bombycis* were notably lower at only 4%, 8%, and 8% in follicular cells, nurse cells, and oocyte cells, respectively (Figure 7C). Analysis of the pathogen load in ovarioles revealed a significantly higher number of pathogen load in nurse cells compared to follicular cells, with only a minor proportion of follicular cells being infected (Figure 7B, D).

### Vg serves as a conserved facilitator in *Nosema* TOT process

**Figure 8.**
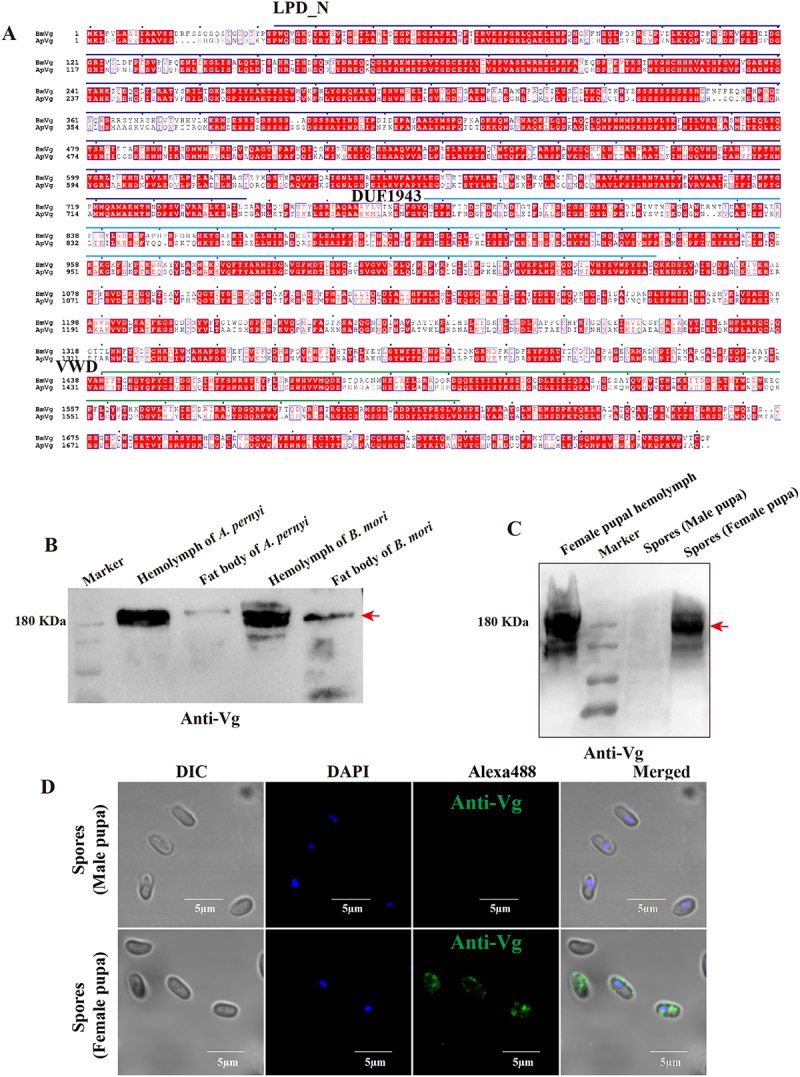
*N. pernyi* bound with ApVg in infected pupae. (A) Multiple sequence alignment of vitellogenin sequences among *B. mori* and *A. pernyi*. The protein domains of Vg were predicted to consist of a vitellogenin N domain (LPD_N), an unknown functional domain (DUF1943), and a von Willebrand domain (VWD). Alignments were performed using ClustalW and visualized using ESPript. Amino acid sequences were obtained from the UniProt database (https://www.Uniprot.org). (B) Detection of polyclonal antibodies against Vg in tissues using western blotting. (C) Western blotting analysis the Vg binding with *N. pernyi* spores. (d) IFA analysis of Vg localization on the surface of *N. pernyi* spores. *N. pernyi* spores isolated from male pupae were incubated with hemolymph of female pupa and labeled with the anti-Vg. Nuclei were stained using DAPI (blue). The green fluorescence indicates the Vg labeled by anti-Vg conjugated with Alexa Fluor 488.

To be successfully transmitted to the offspring, pathogens must employ multiple strategies to cross the barrier and enter the oocyte. Current evidence indicates that pathogens likely exploit the existing pathways utilized by vitellogenin (Vg) [27,28,50,55]. Our previous research demonstrated that *B. mori* Vg plays an essential role in the TOT of *N. bombycis* [24]. Multiple sequence alignment reveals that Vg is highly conserved between *B. mori* and *A. pernyi* (Figure 8A). Western blotting results demonstrate that antibodies against *B. mori* Vg can effectively recognize *A. pernyi* Vg (Figure 8B). To confirm the potential involvement of ApVg in TOT, we investigated the interaction between ApVg and *N. pernyi* spores. Spores were isolated from the infected female and male pupae, and binding of ApVg to the spore surface was determined using IFA and WB. No presence of Vg was detected on the surface of spores isolated from male pupae, whereas it was specifically identified on the surface of spores obtained from female pupae (Figure 8C,D). Similarly, during the infection of *B. mori*, *H. armigera*, and *S. litura* by *N. bombycis*, a binding interaction also occurs between the parasite and the host. Furthermore, we confirmed that *N. bombycis* spores can bind to the recombinant protein of the host Vg functional domain VWD in vitro through co-incubation with spores (Fig S2). Previous studies have systematically validated the interaction between BmVg and *N. bombycis* spore wall proteins [24].

**Figure 9.**
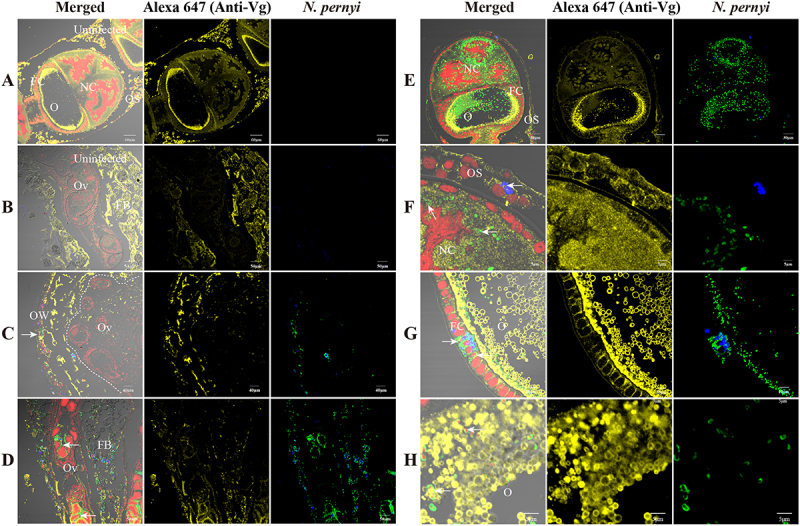
*N. pernyi* is coated with ApVg in infected ovarioles. (A–H) IFA analysis of subcellular colocalization of *N. pernyi* with ApVg in ovary and ovarioles. (A, B) Localization of ApVg in uninfected ovarioles. (C, D) Colocalization of *N. pernyi* and ApVg in ovary at diapause pupal stage. (E, F) Colocalization of *N. pernyi* and Vg in ovariole sheath cells and nurse cells. (G, H) Colocalization of *N. pernyi* and Vg in follicular cells and oocytes. The arrowhead shows *N. pernyi* with the Vg signal; *N. pernyi* spores stained using FB28 (blue); proliferative *N. pernyi* labeled with a rabbit anti-*N. bombycis* polyclonal antibody and conjugated to Alexa Fluor 488 (green); Vg was detected using a mouse anti-Vg polyclonal antibody conjugated to Alexa Fluor 647 (yellow); nuclei were stained using PI (red). OW, ovarian wall; OS, ovariole sheath; FC, follicular cell; O, oocyte; NC, nurse cell.

To verify the potential function of ApVg in the process of TOT, we examined the co-localization of ApVg with parasites. ApVg was found to be localized in various parts of uninfected ovarioles, including the ovariole sheaths, follicular cells, nurse cells, and oocytes (Figure 9A). At the diapause pupal stage, ApVg synthesized by the fat body begins to be transported to the ovarioles and is observed in the ovarian wall and fat bodies (Figure 9B). Furthermore, we observed that when Vg was not largely transported to the ovarioles in the ovary, the parasites could only invade the ovarian wall cells (Figure 9C). More specifically, upon the transport of Vg into the ovariole, we observed parasite infection within this structure (Figure 9D). Additionally, ApVg was observed on the surface of parasites in ovariole sheath (Figure 9E, F), nurse cells (Figure 9F) and follicular cells (Figure 9G). Within the oocyte cytoplasmic region, *N. pernyi* was distributed among yolk granules while being coated with ApVg simultaneously (Figure 9H). Similarly, during the infection of *B. mori*, *H. armigera*, and *S. litura* ovarioles by *N. bombycis*, host Vg was observed on the surface of *N. bombycis* in ovariole cells (Fig S3). These findings suggest that Vg plays a crucial role in facilitating parasites TOT.

**Figure 10.**
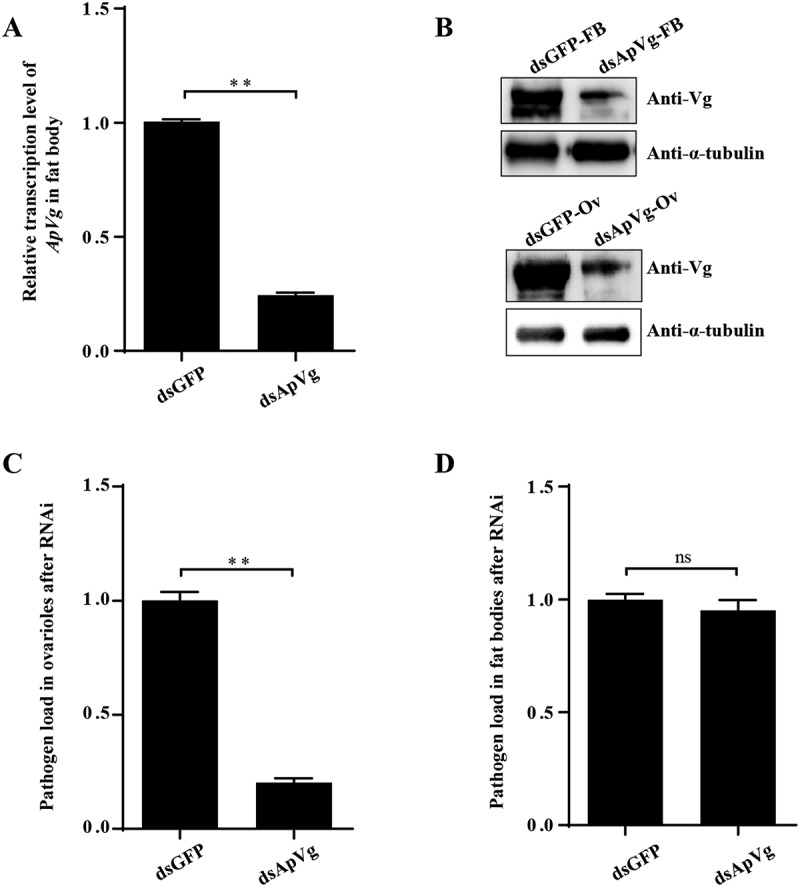
The pathogen load in ovarioles significantly decreased after RNAi of ApVg. (A) The transcription expression of ApVg in the fat body were determined using qPCR with primers of Vg. (B) The protein expression of ApVg in the fat body and ovarioles were determined using western blotting with an antibody against Vg (anti-Vg). FB: fat body; Ov: ovariole. (C) The pathogen load of *N. pernyi* in ovarioles after RNAi Vg. (D) The pathogen load of *N. pernyi* in fat bodies after RNAi Vg. The pathogen load was quantified by the ratio of *Np SSU* copy numbers in the experimental group relative to the control group. Bars represent the means ± SD from three independent experiments. ns, no significant difference; ** *p* < 0.01.

To validate this hypothesis, we employed RNA interference (RNAi) to knock down the expression of ApVg. As a result, there was a significant reduction of approximately 75% in ApVg expression in the fat body of female pupae (Figure 10A). Additionally, the protein expression of ApVg decreased significantly in the fat body and ovarioles (Figure 10B). The pathogen loads in the fat bodies and ovarioles were determined using qPCR with primers for *N. pernyi SSU*. There was a substantial decrease of around 80% in pathogen loads within the ovarioles upon the RNAi (Figure 10C), while it did not suppress infection within the fat bodies (Figure 10D).

## Discussion

Microsporidian TOT has been extensively documented in multiple species [16,30,31,34,36]. These studies usually provide a detailed description of parasite developmental stages, whereas much less is known about the influence of parasites on the course of oogenesis and how and when the oocytes are infected. Our prior research has elucidated key aspects of microsporidian TOT and revealed the key factors for understanding the molecular mechanisms underlying this transmission in *B. mori* [24]. Nevertheless, the precise process and mechanism of *N. pernyi* infecting host oocytes, as well as whether this mechanism is conserved among microsporidia for oocyte entry or if different species employ distinct strategies, remain unclear.

The pébrine caused by *N. pernyi* and *N. bombycis* poses a serious threat to the rearing of silkworms. Similar to the infection of *B. mori* by *N. bombycis*, *N. pernyi* infects all tissues of *A. pernyi* larvae successively after infection, but the specific characteristics and process of their infection of reproductive tissues have not been reported [14]. Our study observed for the first time the process of *N. pernyi* invading the reproductive tissues of *A. pernyi* pupae. We found that this process has both similarities and differences compared with the infection process of *B. mori* by *N. bombycis*. For instance, we observed that *N. pernyi* can invade the ovarian wall cells, as well as the fat body cells surrounding the ovarioles, and the internal ovarioles during the diapause stage. However, *N. bombycis* can only invade the ovarioles after exposure to hemolymph. Moreover, the infection rate of *N. pernyi* in oak silkworms was significantly higher than that of *N. bombycis* in domestic silkworms. We hypothesize that this discrepancy may be attributed to two factors: the unpredictable pupal diapause period in oak silkworms and the varying developmental states of the ovary, both of which could provide parasites with extended opportunities to infect the ovarioles. These findings indicate that despite differences in the timing of ovariole invasion, both *N. pernyi* and *N. bombycis* exhibit comparable infection patterns within the ovarioles of their respective hosts.

By comparing the infection dynamics of *N. pernyi* and *N. bombycis* across different host species, our results provide compelling evidence for the conservation of infection strategies among microsporidian parasites. The infection of oocytes via nurse cells and follicular cells was frequently observed in polytrophic ovarioles. Oocytes in polytrophic ovarioles are connected with nurse cells and surrounded by follicular cells, and encapsulated by sheath cells [26]. Follicular cells associated with nurse cells exhibit greater susceptibility to infection compared to those linked to the oocyte. Therefore, based on the distribution characteristics of parasites in the ovarioles, it can be inferred that most microsporidia primarily infect oocytes through the ovariole sheath, nurse cells, and follicular cells [24,30,31,34,36], rather than via nutrient cords as observed with viruses [25,28,56]. Additionally, the infection rates of *N. pernyi* across different cell types indicate that the parasite preferentially targets nurse cells as a strategic means to gain access to oocytes. Although significant cellular structural changes have been noted following *N. bombycis* infection of ovarioles [24], similar phenomena have not been observed in *N. pernyi* infection. This conserved pattern of infection suggests that microsporidia may utilize similar cellular targets and mechanisms to establish vertical transmission across diverse insect species.

During the process of TOT, the nutrient transport pathway used by Vg entering the oocytes was utilized by the parasites, thereby enabling the parasites to invade the ovarioles. For instance, *N. bombycis* directly interacts with Vg during TOT process not only in *B. mori* but also in *S. litura* and *H. armigera* [24]. Similarly, our study demonstrates that *N. pernyi* binds with ApVg during the process of ovariole infection. When Vg expression is downregulated, the infection of ovarioles by *N. pernyi* is significantly reduced. More specifically, ApVg is already highly expressed during diapause when *N. pernyi* can invade the ovarioles, which are still enveloped by fat bodies. In contrast, Vg begins to be expressed in high quantities around the third day of the pupal stage in *B. mori*, at which point the ovarioles have already exposed to the hemolymph, allowing *N. bombycis* to invade the ovarioles. In addition, this conserved transportation system has been extensively exploited by a wide range of pathogens and endosymbiotic microorganisms for vertical transmission [27,28,42,50,55,56]. The parasites utilize host nutrient transportation system by binding with Vg, thereby facilitating its access to host oocytes. Vg is highly conserved and serves not only as a critical nutrient reserve for embryonic development but also as an important factor involved in pathogen recognition [46–48,57]. Its conserved nature and essential biological function may facilitate the convergent evolution of parasites, enabling them to exploit Vg for TOT. It will be interesting to investigate whether nutrient transportation system plays a vital role in microsporidian TOT.

Microsporidia undergo multiple life stages, culminating in spores [35]. *N. pernyi* in various forms were found in ovariole sheath cells and follicular cells, whereas only proliferative stages were found in nurse cells and oocytes. These findings suggest that *N. pernyi* likely penetrate the oocyte membrane barrier mainly in proliferative stages instead of in mature spores. The gap between follicular cells, nurse cells, and oocytes after infection with *N. bombycis* was obviously changed, and a vesicular structure was formed to facilitate the invasion of oocytes [24]. However, no significant changes in cell structure have been observed after *N. pernyi* infection. In particular, after invading nurse cells and oocytes, the *N. pernyi* and *N. bombycis* exhibit distinct morphological and distributional characteristics, indicating that their infection and proliferation strategies are different. This further prompts us to consider whether the parasite adopts different infection strategies for different cell types within the ovarioles. Therefore, the characteristics of the parasite at the infection interface warrant further detailed observation and analysis.

The components of the pathogen play a critical role in the establishment of vertical transmission. Pathogens often exploit either host-derived or intrinsic mechanisms to create favorable conditions for breaching physiological barriers and entering oocytes [28,43,50,58,59]. For instance, RGDV can utilize its own non-structural protein Pns11 to break through the TOT barrier [43]. RSV nucleocapsid protein (NP) binds to host Vg, and then uses the Vg transport pathway to enter the developing ovaries [28,50]. In the ovaries, the interaction of NP with Rab1 maintains a balance between vector reproduction and viral vertical transmission [60]. In the context of *N. bombycis* TOT in *B. mori*, the interplay between Vg and the spore wall proteins (SWP) is vital [24]. Co-localization results indicated that SWP12, SWP26, and SWP30 could also interact with the Vg of *S. litura* and *H. armigera* on the spore wall (Fig S2C). Moreover, the SWP12 and SWP30 exhibit high conservation between *N. bombycis* and *N. pernyi* (Fig S4), suggesting potential conserved mechanisms in their TOT processes. Therefore, we speculate that the binding of ApVg to *N. pernyi* spores may also be mediated by SWPs, and the SWPs of *N. pernyi* may also play an important role in its TOT process. Further investigation into the molecular and cellular processes underlying these differences may provide valuable insights into the evolutionary adaptations of microsporidia for successful vertical transmission in diverse host environments.

Studies have demonstrated a strong association between the virulence or pathogenicity of pathogens and their modes of transmission [61–64]. Microsporidia exhibit diverse transmission modes and varying degrees of pathogenicity in different animal hosts [16]. Horizontal transmission represents the primary route of transmission utilized by most microsporidia, which exhibit strong pathogenicity and may play a significant role in regulating host population size [62,65]. A small number of microsporidia can only infect hosts through the TOT route, such as *Nosema empoascae* and *Nosema granulosis* [32,34]. These microsporidia are frequently present in low burden and cause little pathogenicity. At the same time, the TOT parasite can regulate host population dynamics through sex ratio distortion, leading to feminization or male killing [16,66]. Many microsporidia exhibit both horizontal and transovarial transmission, such as *N. bombycis* and *N. pernyi*, which may have a stronger regulatory effect on the host population. When pathogen load in the host is sufficiently high, it may result in host mortality and promote horizontal transmission among conspecifics. Conversely, TOT occurs at lower pathogen loads and has the potential to alter the offspring sex ratio.

## Conclusions

This study illustrated that the TOT of *Nosema* species, *N. pernyi* and *N. bombycis*, shows both conserved and species-specific strategies in different hosts. Both parasites follow a stepwise invasion pathway through ovarian tissues, targeting ovariole sheath cells, follicular cells, nurse cells, and ultimately oocytes. However, *N. pernyi* exhibited higher infectivity in ovarian tissues during diapause compared to *N. bombycis*, and their proliferation patterns within oocytes differed significantly. We also discovered that host Vg plays a critical role in facilitating microsporidian TOT across both silkworm species, as knocking down Vg expression dramatically reduced parasite loads in the ovaries. These findings demonstrate that *N. pernyi* and *N. bombycis* employ a conserved, stepwise TOT mechanism in silkworms, despite species-specific differences in early infection dynamics. These findings provide novel insights into the evolutionary conservation of TOT mechanisms among microsporidian parasites and highlight Vg as a potential molecular target for disease intervention, and establishes a framework for understanding TOT across economically significant Lepidoptera. This research enhances our understanding of parasite–host interactions and offers new strategies to control these infections.

## Supplementary Material

Figure S1.tif

Table S1.doc

Figure S4.tif

Figure S2.tif

Raw data for graph.xlsx

Figure S3.tif

## Data Availability

The data that support the findings of this study are openly available in figshare at https://doi.org/10.6084/m9.figshare.30147481, reference number [67].
