# Supplementary figures and images for: Unveiling the conserved mechanism of microsporidian vertical transmission: A comparative study of Nosema infection across host species
Source: Virulence. 2025 Dec 23;17(1):2609384. doi: 10.1080/21505594.2025.2609384 (PMC12758177; doi:10.1080/21505594.2025.2609384)

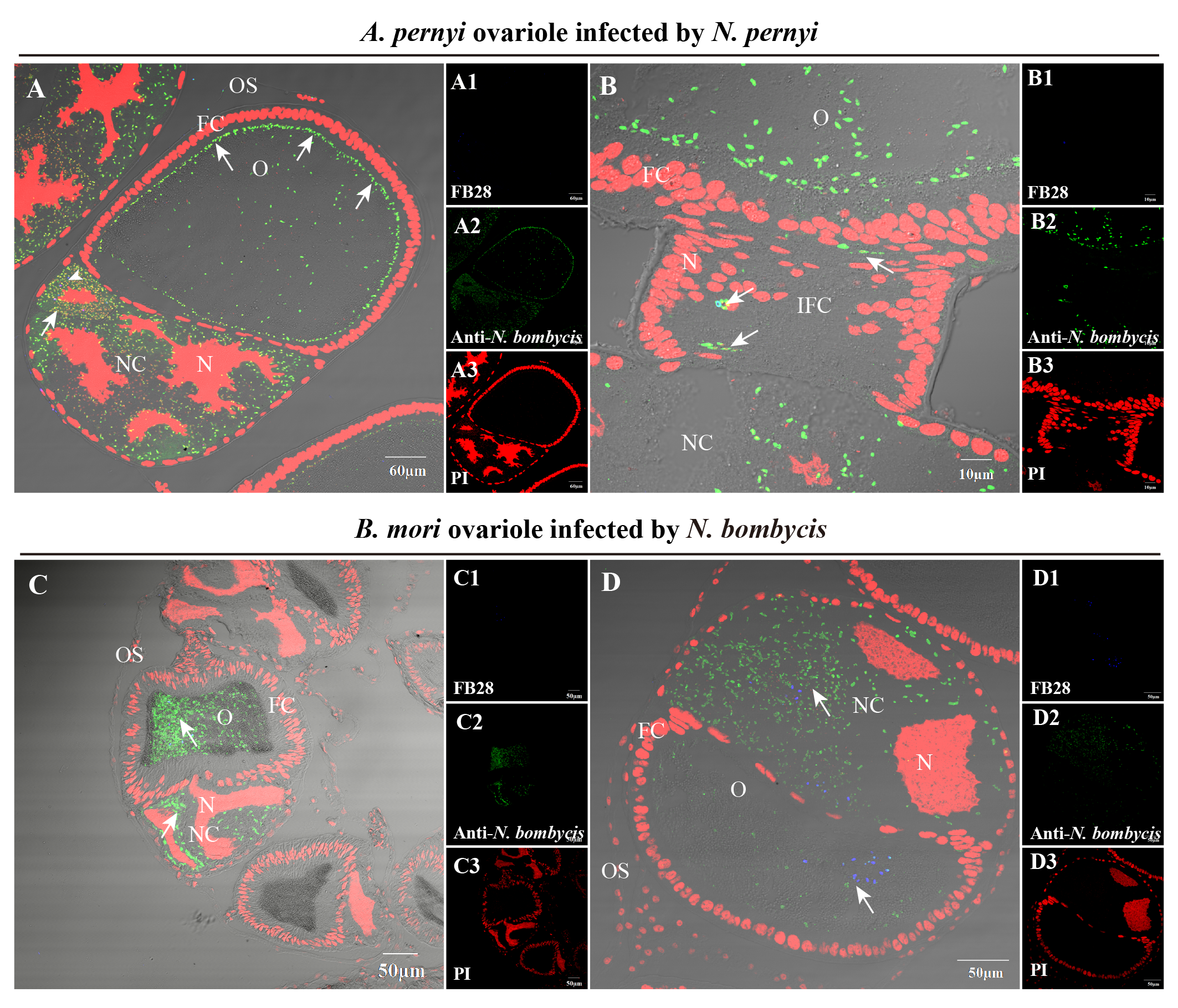

Supplement: Figure S1.tif [file KVIR_A_2609384_SM1757.tif]

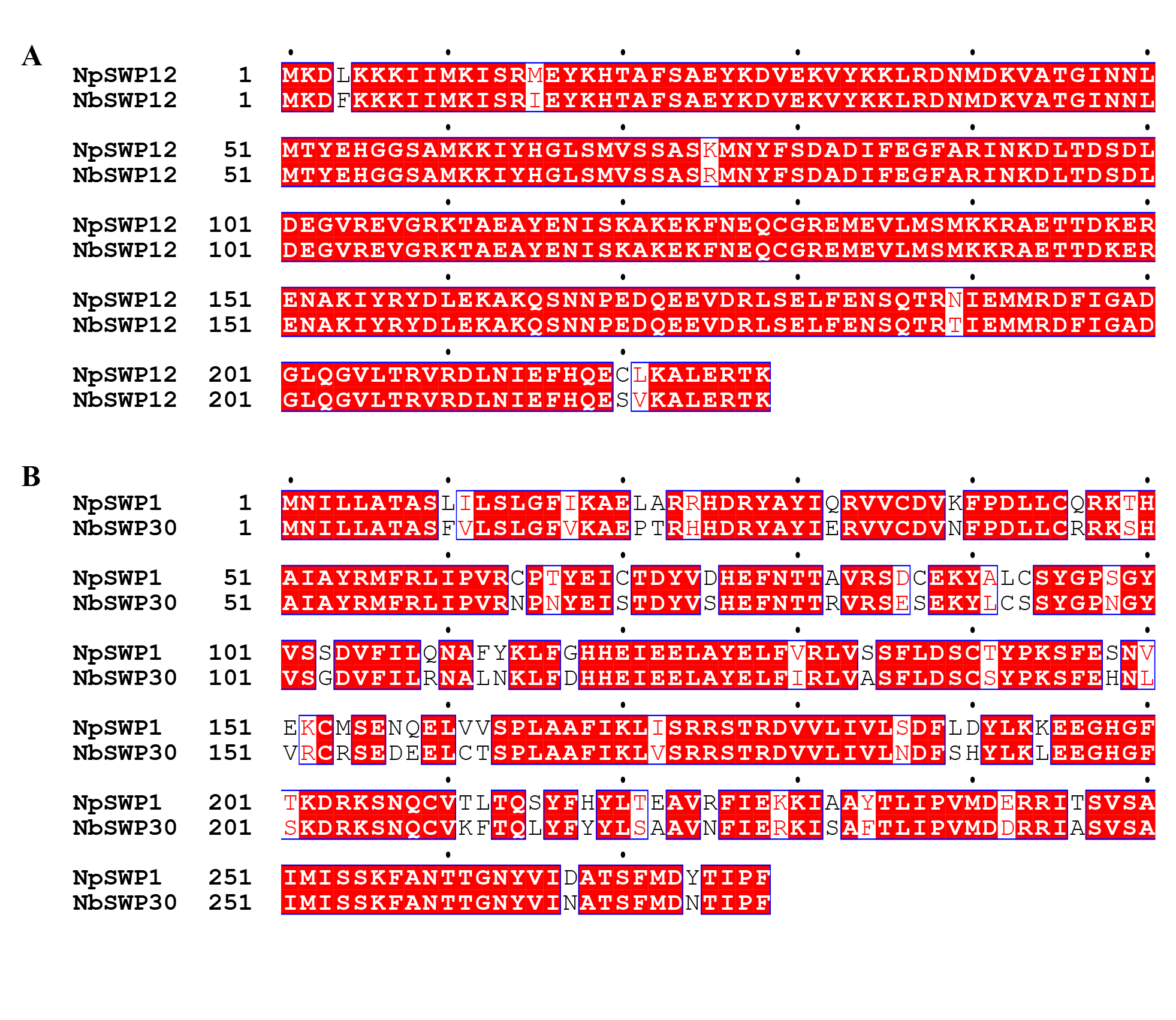

Supplement: Figure S4.tif [file KVIR_A_2609384_SM1755.tif]

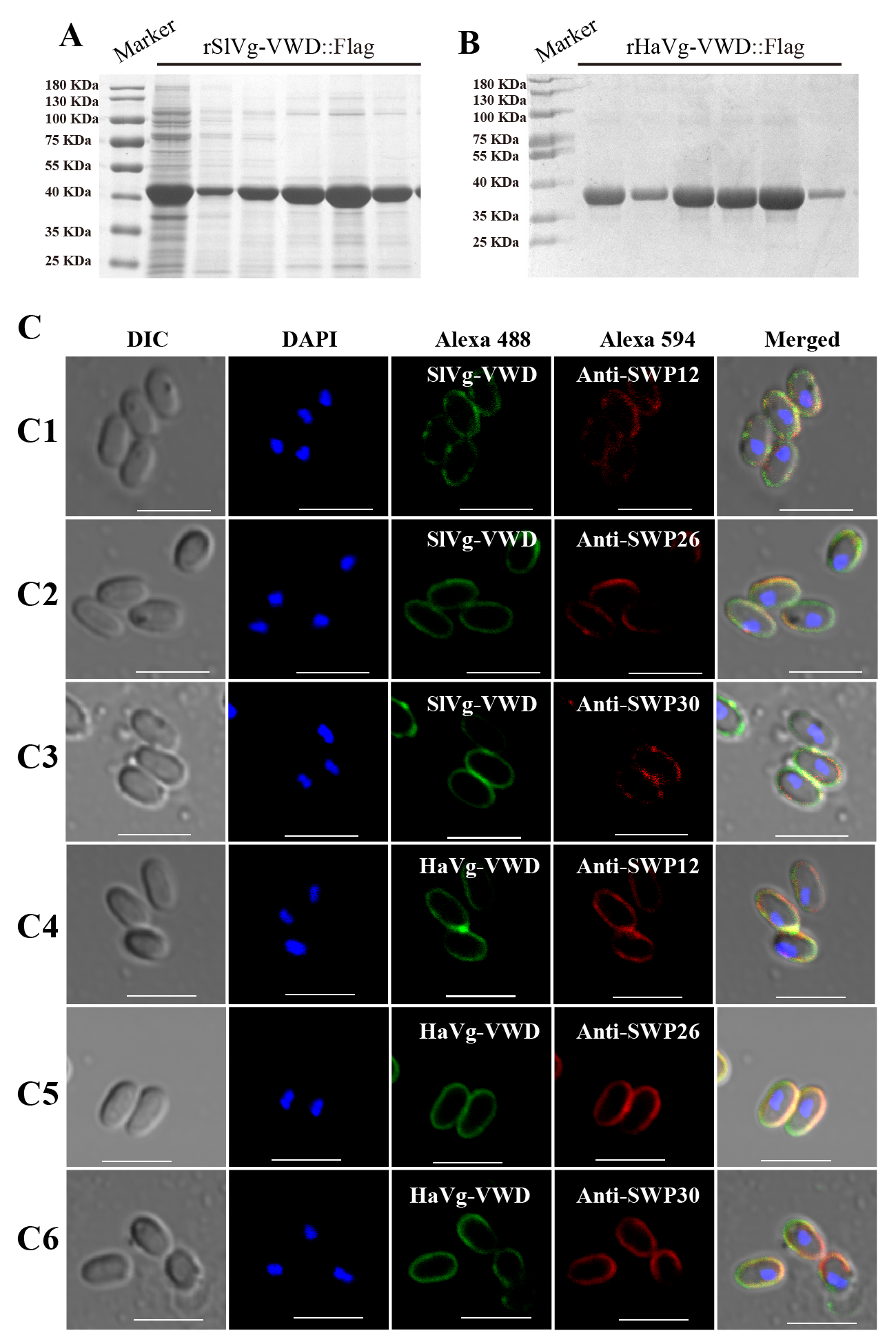

Supplement: Figure S2.tif [file KVIR_A_2609384_SM1754.tif]

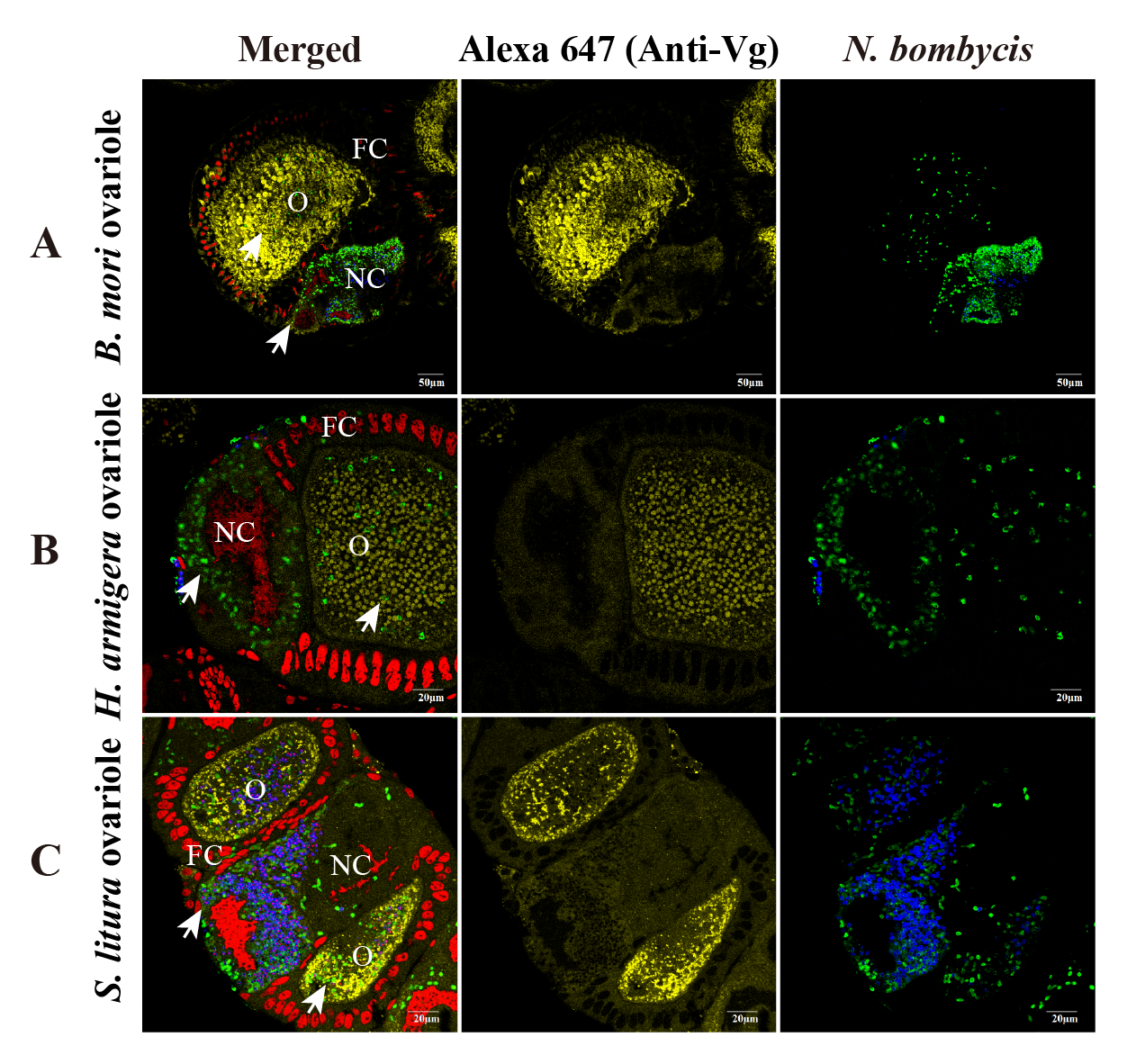

Supplement: Figure S3.tif [file KVIR_A_2609384_SM1752.tif]
