# Supplementary material for: Unveiling the conserved mechanism of microsporidian vertical transmission: A comparative study of Nosema infection across host species
Source: Virulence. 2025 Dec 23;17(1):2609384. doi: 10.1080/21505594.2025.2609384 (PMC12758177; doi:10.1080/21505594.2025.2609384)
Supplement: Table S1.doc [file KVIR_A_2609384_SM1756.doc]

S1 Table Primers used in this study.

| Purpose | Construct | Primer sequences | | |
| --- | --- | --- | --- | --- |
|  |  | Forward |  | Reverse |
| For construction of sequencing vector | pMD19-T-APVg | 5'- GGATCCATGCAAGACGGAAAGGTTTAC -3' |  | 5'- GAATTCTTAAATTCCAGATTTAAGTGC-3' |
|  | pMD19-T-NpSSU | 5'-CACCAGGTTGATTCTGCC -3' |  | 5'-TTATGATCCTGCTAATGGTTC-3' |
| dsRNA assay | T7-GFP | 5'-TAATACGACTCACTATAGGGAGATGCTTCAGCCGCTACCC-3' |  | 5'-TAATACGACTCACTATAGGGAGATCCAGCAGGACCATGTGAT  -3' |
|  | T7-ApVg | 5'-TAATACGACTCACTATAGGGAGAAGATCCAAGGTGAAGAAGCTG- 3' |  | 5'- TAATACGACTCACTATAGGGAGATTCTGACTGGCCAGAACAAAA -3' |
| RT-qPCR assay | q-APVg | 5'-GAAGGTGCTGAATGGACTGGTA-3' |  | 5'-CTCTGGTTTCTCCCATTCAGCT- 3' |
|  | q-NP-SSU | 5'-AGGACGTAAGCTAGAGGATCGA-3' |  | 5'-TACCCCGCGTTGAGTCAAATTA-3' |
|  | q-Ap18S | 5'-CGATCCGCCGACGTTACTAC-3' |  | 5'-GTCCGGGCCTGGTGAGATT-3' |
